# Supplementary material for: The Novel Role of Zfp296 in Mammalian Embryonic Genome Activation as an H3K9me3 Modulator
Source: Int J Mol Sci. 2023 Jul 12;24(14):11377. doi: 10.3390/ijms241411377 (PMC10379624; doi:10.3390/ijms241411377)
Supplement: Supplementary file 1 [file ijms-24-11377-s001.zip › Supplement figtures_20230629233457.pdf]

S1

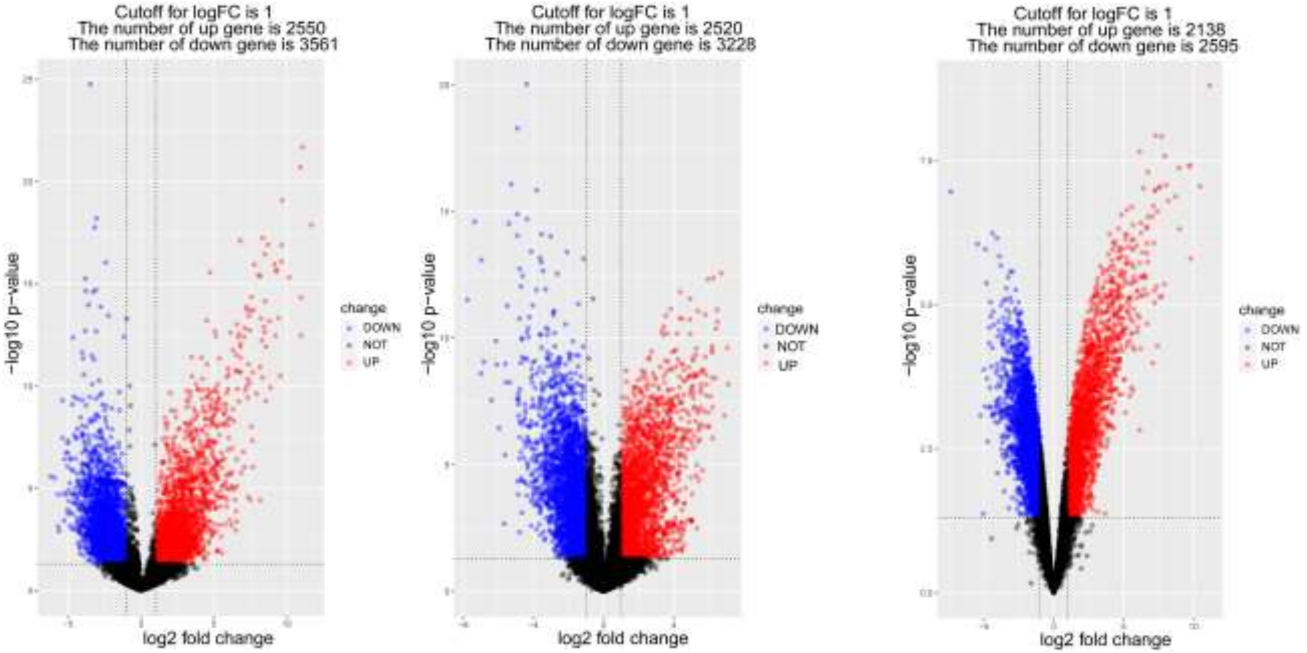

Figure S1. RNA-seq analysis of human, mouse and goat early embryos(padj < 0.05 |log2(fold change)| ≥ 1).

S2

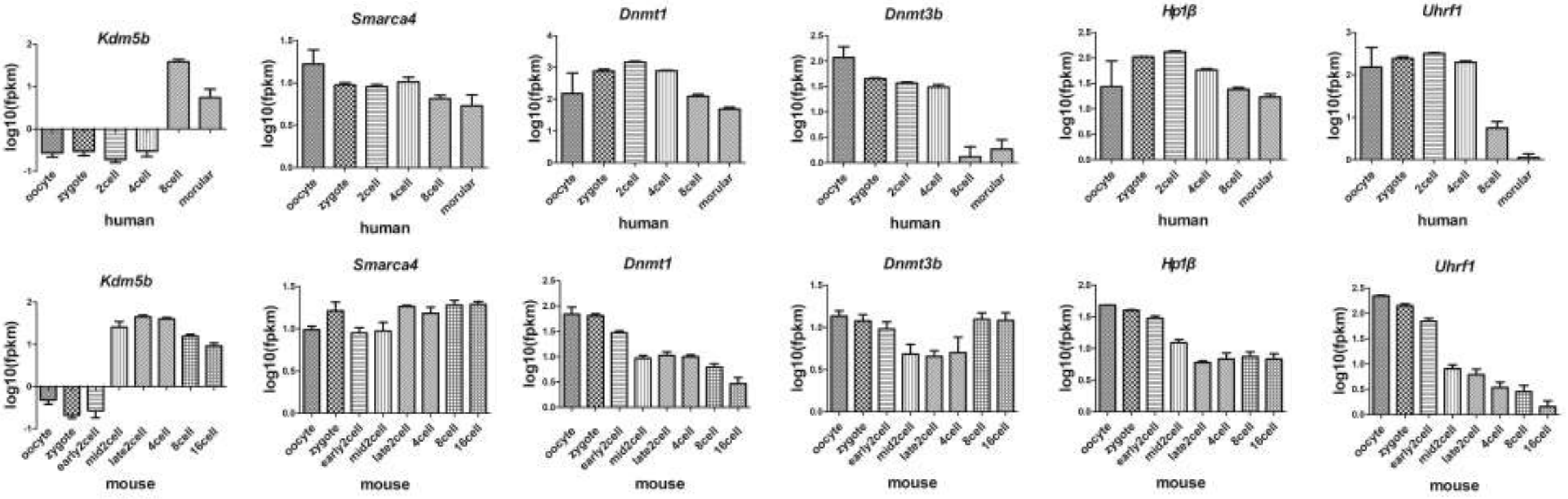

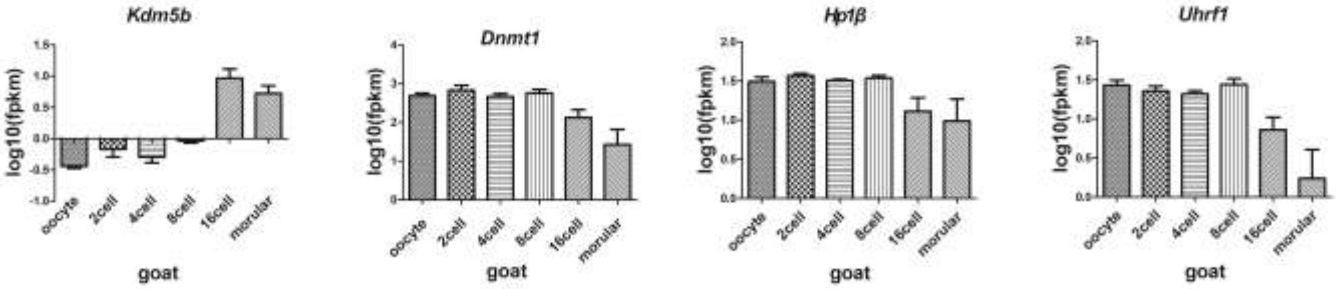

Figure S2.The expression of *Kdm5b,Smarca4,Dnmt1, Dnmt3b,Hp1β* and *Uhrf1* in human, mouse and goat embryos.

S3

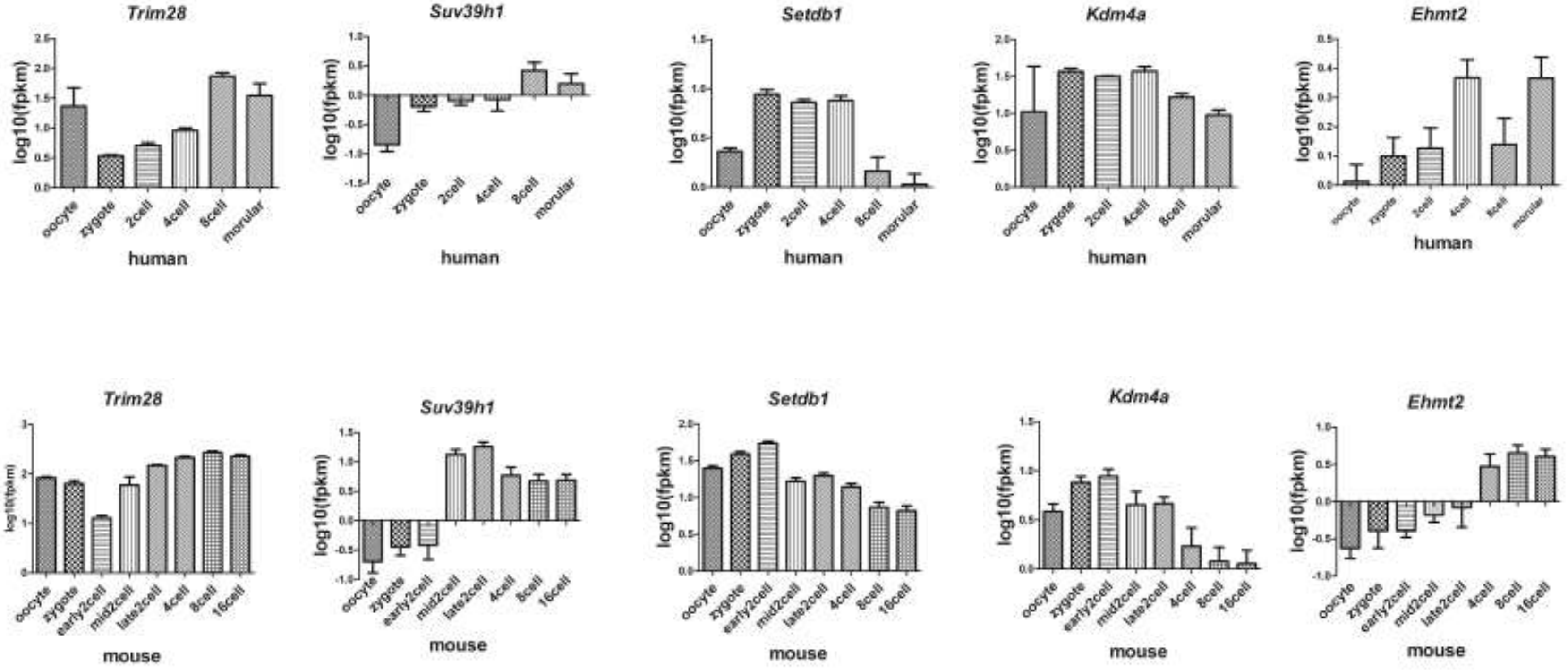

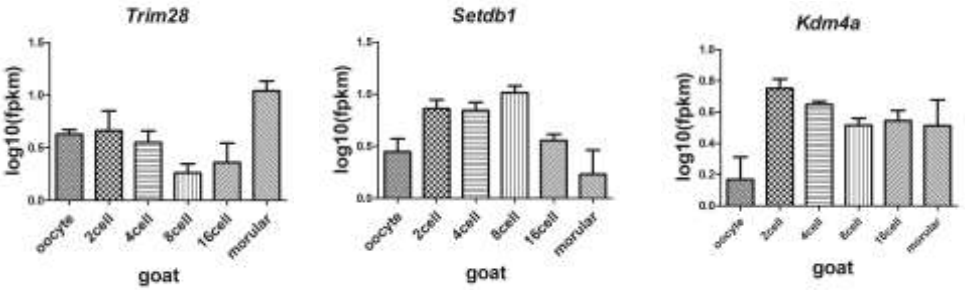

Figure S3.The expression of *Trim28*,*Suv39h1*,*Setdb1*,*Kdm4a* and *Ehmt2* in human,mouse and goat embryos.

S4

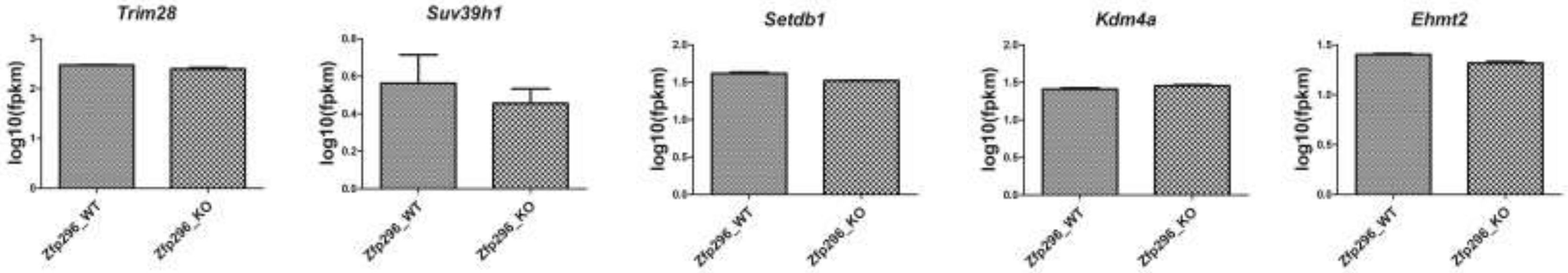

Figure S4.The expression of *Trim28*,*Suv39h1*,*Setdb1*,*Kdm4a* and *Ehmt2* in *Zfp296* KO ESCs.

S5

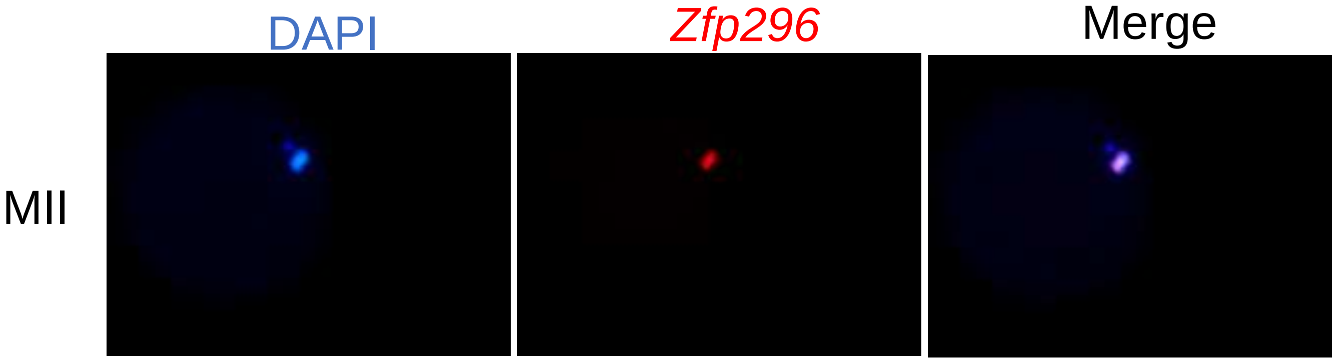

S5

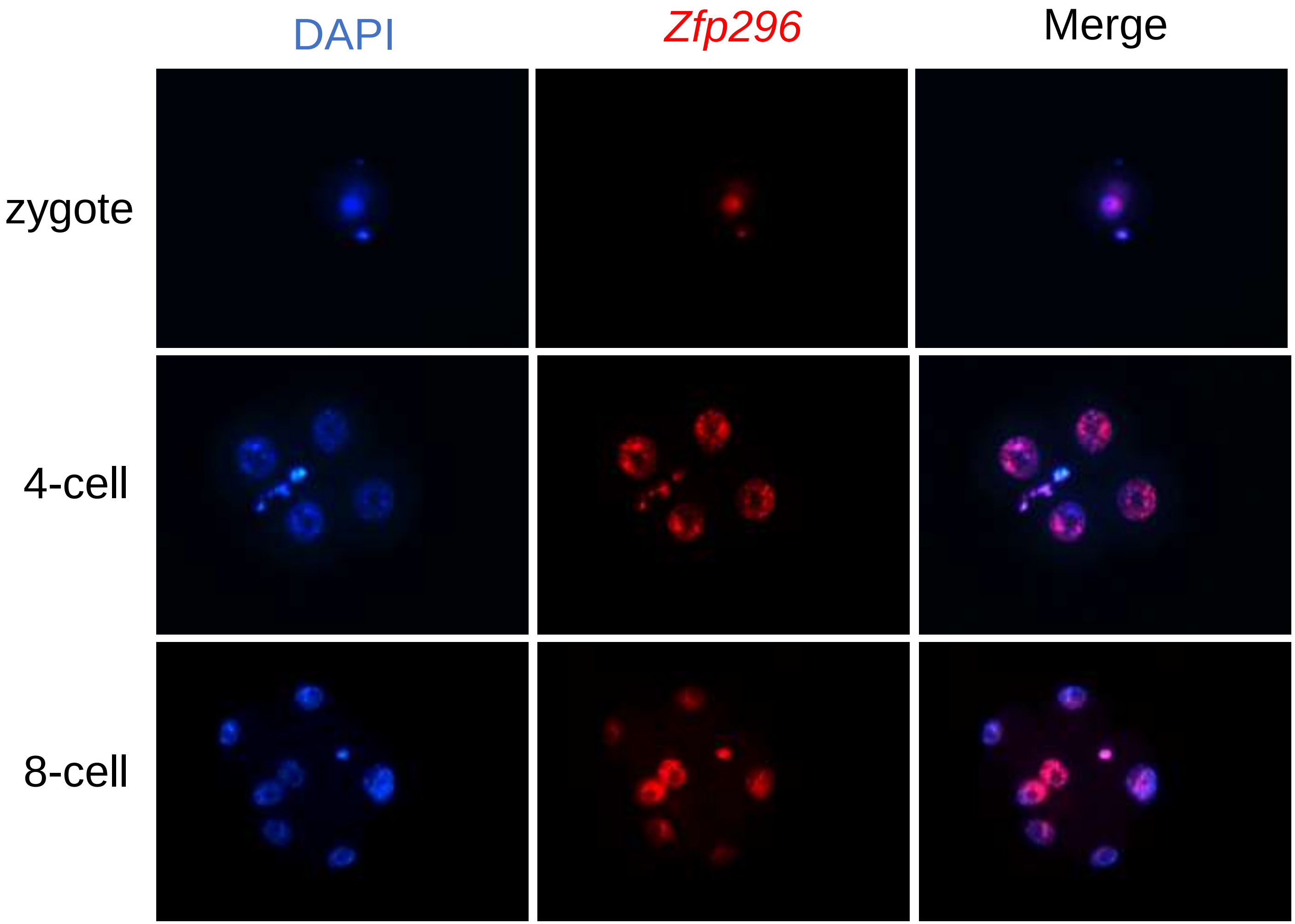

Figure S5. The distribution of *Zfp296* in MII and other stages of early embyros.
